# Intraoperative transfusion practices and perioperative outcome in the European elderly: A secondary analysis of the observational ETPOS study

**DOI:** 10.1371/journal.pone.0262110

**Published:** 2022-01-04

**Authors:** Linda Grüßer, András Keszei, Mark Coburn, Rolf Rossaint, Sebastian Ziemann, Ana Kowark, the ETPOS Study Group

**Affiliations:** 1 Department of Anaesthesiology, University Hospital RWTH Aachen, Aachen, Germany; 2 Center for Translational & Clinical Research Aachen (CTC-A), Medical Faculty RWTH Aachen University, Aachen, Germany; 3 Department of Anaesthesiology and Intensive Care Medicine, University Hospital Bonn, Bonn, Germany; Brown University Warren Alpert Medical School, UNITED STATES

## Abstract

The demographic development suggests a dramatic growth in the number of elderly patients undergoing surgery in Europe. Most red blood cell transfusions (RBCT) are administered to older people, but little is known about perioperative transfusion practices in this population. In this secondary analysis of the prospective observational multicentre European Transfusion Practice and Outcome Study (ETPOS), we specifically evaluated intraoperative transfusion practices and the related outcomes of 3149 patients aged 65 years and older. Enrolled patients underwent elective surgery in 123 European hospitals, received at least one RBCT intraoperatively and were followed up for 30 days maximum. The mean haemoglobin value at the beginning of surgery was 108 (21) g/l, 84 (15) g/l before transfusion and 101 (16) g/l at the end of surgery. A median of 2 [1–2] units of RBCT were administered. Mostly, more than one transfusion trigger was present, with physiological triggers being preeminent. We revealed a descriptive association between each intraoperatively administered RBCT and mortality and discharge respectively, within the first 10 postoperative days but not thereafter. In our unadjusted model the hazard ratio (HR) for mortality was 1.11 (95% CI: 1.08–1.15) and the HR for discharge was 0.78 (95% CI: 0.74–0.83). After adjustment for several variables, such as age, preoperative haemoglobin and blood loss, the HR for mortality was 1.10 (95% CI: 1.05–1.15) and HR for discharge was 0.82 (95% CI: 0.78–0.87). Pre-operative anaemia in European elderly surgical patients is undertreated. Various triggers seem to support the decision for RBCT. A closer monitoring of elderly patients receiving intraoperative RBCT for the first 10 postoperative days might be justifiable. Further research on the causal relationship between RBCT and outcomes and on optimal transfusion strategies in the elderly population is warranted. A thorough analysis of different time periods within the first 30 postoperative days is recommended.

## Introduction

At the beginning of 2018 the elderly aged 65 years and older accounted for 19.7% of the total population in Europe. Their share is projected to increase to 28.5% in 2050 [1]. This demographic development suggests a dramatic growth in the number of elderly patients undergoing surgical procedures.

In general, anaemia in older patients is common and prevalence rises with age: It was estimated that in the United States more than 10% of adults aged 65 years and older and more than 20% of adults aged 85 years and older present with anaemia as defined by the World Health Organization (WHO) [2]. Pre-operative anaemia was identified to be independently associated with an increased risk of 30-day mortality in non-cardiac surgical patients [3].

In the perioperative setting, where pre-operative anaemia might be accompanied by surgical blood loss, red blood cell transfusions (RBCT) are commonly applied [4]. Advanced age has repeatedly been identified as a patient variable associated with perioperative RBCT [5]. A retrospective cohort study involving 20,930 patients at a tertiary medical centre in the U.S., revealed that older patients had 62% greater odds of receiving RBCT in the perioperative period in comparison to younger patients [6]. Previous findings revealed the majority of RBCT is given to patients who are aged 65 years and older [7]. Importantly, demographic changes are also likely to affect supply and demand of RBCT, as the increase of the older age groups is expected to come along with a decrease of the younger potential donor population [7].

Despite international transfusion guidelines, variability in perioperative transfusion rates persists and there is a scarcity of data on the overall transfusion practice in the elderly surgical cohort [8–11], in particular in the European population. As blood transfusions carry risks, are costly and their supply is limited, patient blood management principles are imperative [12, 13]. Overall, guidelines recommend a restrictive transfusion regime, which has been proven safe in most clinical settings [14–18]. On the other hand, some studies have favoured a liberal regime [11, 19, 20]. It has been discussed whether patients with cardiovascular diseases are less capable of tolerating anaemia. Based on this assumption, it is argued whether liberal transfusion practices might be beneficial for this risk group [20, 21]. Compensatory mechanisms might also be reduced in old and frail patients. A recent meta-analysis confirmed favourable outcomes of a liberal transfusion regime in older patients [11]. Up until now, evidence with regard to the optimal haemoglobin (Hb) threshold for perioperative RBCT in particular for the elderly patients is lacking and subject of ongoing research (Liberal Transfusion Strategy in Elderly Patients [LIBERAL], NCT03369210).

The European Transfusion Practice and Outcome Study (ETPOS) study was a prospective multi-centre study evaluating general intraoperative transfusion practices throughout Europe [22]. The present secondary analysis and substudy of the ETPOS data is the first study that describes perioperative transfusion practice and related outcomes in specifically the elderly European population undergoing surgical procedures. We assessed triggers for transfusion and the association of intraoperative RBCT with mortality and discharge from hospital. Our findings might have an important impact on our daily clinical practice.

## Methods

### Study design

We performed a secondary analysis of the prospective observational multicentre ETPOS study [22]. The original study was registered with ClinicalTrials.gov (NCT01604083). The detailed study design that resulted in the present dataset was published previously [22]. The objective of this secondary analysis and subgroup analysis was to assess existing transfusion practices and related perioperative outcomes in specifically the elderly population in Europe. Though not pre-specified [22], the decision to perform this study based on the lack of data on transfusion practices particularly in the elderly patient and was planned before the present data analysis. This analysis is reported in concordance with the STROBE statement (S1 Checklist).

### Ethics

Following approval from the ethics or regulatory board of each participating centre and according to the regulatory requirements, a postoperative written informed consent was obtained or waived for each included patient in the main ETPOS study [22]. A separate ethical approval for this secondary analysis was not required.

### Setting and participants

The original study analysed 5803 patients in 126 European centres during a continuous three-month period in 2013 [22]. Adult patients who underwent an elective non-cardiac surgery and received at least one RBCT intraoperatively were included [22]. This manuscript presents a subgroup analysis specifically for the elderly in Europe: The specific inclusion criterion for this secondary analysis was the minimum age of 65 years. All data were originally prospectively collected. Each patient was followed up until discharge or death for 30 days maximum.

### Data collection

As previously described in detail, data on patient-, surgery- and transfusion-related characteristics were collected and managed with OpenClinica open source software, version 3.2. (Copyright© OpenClinica LLC and collaborators, Waltham, MA, USA, www.OpenClinica.com) [22]. An overview of the study protocol is also provided on the ETPOS website [23]. In brief, information on patient demographics, ASA physical status, duration of anaesthesia, type of surgery, usage of point of care monitoring, transfusion-related laboratory values at the beginning of surgery, just before transfusion of the first RBCT and at the end of surgery, intraoperative fluid and blood product management as well as reasons for the initial transfusion of at least one RBCT were collected. Follow-up data of this study comprised length of hospital stay (LOS), intensive care unit (ICU) length of stay, ventilator hours and mortality until 30 postoperative days maximum.

### Outcome measures

Our primary aim was to assess existing transfusion practices. We analysed the amount of RBCT given, transfusion triggers and transfusion-related laboratory values. Pre-operative anaemia was defined as Hb <120 g/l for women and <130 g/l for men according to the WHO classification [24]. Further outcome measures were mortality within 30 days after surgery, LOS and ICU length of stay. A descriptive subgroup analysis was performed for the following three age groups: 65–74, 75–84 and 85–100 years. We hypothesized that the amount of intraoperative RBCT is independently associated with 30-day mortality and discharge from hospital.

### Bias

Given the observational character of this study, ETPOS aimed to minimise selection bias and provide more generalizable results by consecutive recruitment of all eligible patients during the three months period.

### Statistical analysis

The statistical analysis of patients aged 65 years and older was carried out on the basis of the already cleaned and closed ETPOS database [22]. Statistical analysis was performed using R Core team 3.5.0 (R Foundation for Statistical Computing, Vienna, Austria). With regard to descriptive statistics of patient-, surgery and transfusion-related characteristics and follow-up data, categorial variables are presented as number (% of total sample) and continuous variables as median [IQR] and mean (SD) respectively. Missing information is listed for each variable if applicable.

Descriptive statistics of RBCT units, including mean, SD, median, IQR, and proportion of patients receiving <2,2–4,>4 units RBCT, were calculated overall and by surgery types, and study centers. The association between the amount of RBCT during surgery and discharge from hospital, as well as death, within 30 days was analysed using Cox proportional hazards model. The models were built on the basis of complete cases. To model the relative hazard of outcome, three models were considered for both outcomes: The first model was unadjusted. The second model was adjusted for age (years), Hb before surgery (g/l), sex (male/female), ASA (1 and 2; 3; 4 and 5), presence of comorbidities (yes/no), cancer surgery (yes/no), stay in intensive care unit (ICU) (no/yes-planned/yes-not planned), type of surgery (8 categories) and random intercepts for center. The third model was additionally adjusted for blood loss (<500ml, 500-1000ml, ≥1000ml). Centers were included in the models as random intercepts assuming a Gaussian distribution. These models were estimated using penalized partial likelihood [25] and the Akaike’s information criteria were calculated. Since model verification suggested that the assumption of proportional hazards for RBCT effect might be unreasonable, time-varying RBCT effects were modeled using three time periods (0–10 days, >10–20 days, >20–30 days after surgery).

## Results

The ETPOS database provided information on 3157 patients who were 65 years or older. One patient was excluded as data on RBCT were lacking. Seven further patients with extremely low Hb values <50g/l prior to surgery were excluded from analysis as this contradicts the principle of an elective surgery. In total, 3149 patients from 123 centres were analysed (S1 File).

The demographic patient data are presented in Table 1. The mean age of this study population was 75.5 (7.2) years. The largest age group was between 65–74 years old. The majority of patients was classified as ASA 3 or higher. Transfusion relevant comorbidities were mostly cardiovascular diseases. Most common procedures were orthopaedic or general surgeries. 37% of surgeries were cancer-related. The median surgical duration was 3.2 [3.2 to 4.8] hours.

**Table 1:** Patient and surgery characteristics.

	n = 3149
Patient age; y	75.5 (7.2)
65 to 74	1534 (49%)
75 to 84	1222 (39%)
85 to 100	393 (12%)
Sex	
female	1631 (52%)
male	1518 (48%)
Height; cm*	166.3 (9.6)
Weight; kg*	72.5 (15.6)
ASA Class*_:_	
ASA 1, n (%)	34 (1%)
ASA 2, n (%)	863 (27%)
ASA 3, n (%)	1668 (53%)
ASA 4, n (%)	538 (17%)
ASA 5, n (%)	43 (1%)
Anaemia according to WHO classification*	2311 (73%)
female^†^	1136 (70%)
male^†^	1175 (77%)
Transfusion relevant comorbidities*	1950 (62%)
Gastro-intestinal	136 (4%)
Haematological	166 (5%)
Pulmonary	193 (6%)
Other	207 (7%)
Renal	223 (7%)
Not specified	607 (19%)
Cardiovascular	1077 (34%)
Type of surgery	
Head and Neck	44 (1%)
Thoracic	58 (2%)
Other	66 (2%)
Gynaecological	119 (4%)
Neurological	129 (4%)
Vascular	424 (13%)
Orthopaedic	1086 (34%)
General	1223 (39%)
Cancer-related surgery	1153 (37%)
Surgery duration (hours)*	3.2 [2.1 to 4.8]

Values are presented as a number (% of total sample) or median [IQR] or mean (SD).

*Missing information on 765 patients concerning height, 380 patients concerning weight, 3 patients concerning the ASA classification, 71 patients concerning Hb-value before surgery, 2 patients concerning transfusion relevant comorbidities and 1006 patients concerning the surgery duration.

^†^% of female and male refer to number of total females and males respectively.

Transfusion-related characteristics are specified in Table 2. The median intraoperative blood loss was 700 [400 to 1350] ml. At the beginning of surgery, the mean Hb was 108 (21) g/l (median Hb 106 [93 to 123] g/l) and decreased to 84 (15) g/l (median Hb 83 [75 to 92] g/l) before transfusion. A median of 2 [1–2] units of RBCT were administered. The amount of RBCT by type of surgery is presented in S1 Fig. An Hb trigger was present in almost 50% of cases and the mean reported threshold limit was 83 (13) g/l. A physiological trigger was present in 66% of patients with hypotension being the most frequent. About 30% of patients showed the combination of an Hb trigger and a physiological trigger or a comorbidity trigger, respectively. Comorbidity alone as a transfusion trigger was reported in almost 10% of patients. At the end of surgery, the mean Hb was 101 (16) g/l (median Hb 100 [91 to 111] g/l).

**Table 2:** Transfusion related characteristics.

	n = 3149
**Blood loss**	
Intraoperative blood loss; ml*	700 [400 to 1350]
Patients with blood loss ≥ 10l	16 (<1%)
**Laboratory values**	
At the beginning of surgery:	
Hb; g/l*	108 (21)
INR*	1.1 (0.5)
aPTT; sec.*	31.9 (14.2)
Fibrinogen; mg/dl*	421.7 (163.2)
Platelets; 1000/ml*	260.2 (120.2)
Before transfusion:	
Hb; g/l*	84 (15)
INR*	1.4 (0.8)
aPTT; sec.*	41.8 (39.6)
Fibrinogen; mg/dl*	324.9 (172.2)
Platelets; 1000/ml**	208.1 (116.3)
At the end of surgery:	
Hb; g/l*	101 (16)
INR*	1.5 (6.4)
aPTT; sec.*	37.6 (26.2)
Fibrinogen; mg/dl*	317.0 (157.7)
Platelets; 1000/ml*	200.8 (111.7)
**Point of care device**	
Point of care device used*	454 (14%)
**RBCT**	
Units of intraoperative RBCT	2 [1 to 2]
Patients with >25units	7 (<1%)
In age group 65–74 years	2 [1 to 3]
In age group 75–84 years	2 [1 to 2]
In age group 85–100 years	2 [1 to 2]
**Intravenous fluids**	
Crystalloids administered*	3116 (99%)
Crystalloids; ml*	2000 [1000 to 3000]
Colloids administered*	1991 (63%)
Colloids; ml	500 [500 to 1000]
**Reasons for transfusion of first RBCT**	
Triggers present:	
Hb trigger present*	1550 (49)
• Hb threshold limit g/l*	83 (13)
Physiological trigger present*	2080 (66%)
• ScO2 decline	41 (1%)
• SvO2 decline	55 (2%)
• Significant ECG changes	96 (3%)
• Arrythmia	168 (5%)
• Increase in lactate	174 (6%)
• Acidosis	178 (6%)
• Other	183 (6%)
• Tachycardia	622 (20%)
• No physiological trigger specified	692 (22%)
• Hypotension	1179 (37%)
Comorbidity trigger present*	1950 (62%)
Bleeding trigger present*	821(26%)
Combinations of triggers	
• Hb trigger and physiological trigger present*	870 (28%)
• Hb trigger and comorbidity trigger present*	977 (31%)
• Hb trigger and bleeding trigger present*	56 (2%)
Only one trigger present	
• Only Hb trigger present*	277 (9%)
• Only physiological trigger present*	520 (17%)
• Only comorbidity as trigger present*	227 (7%)
• Only bleeding trigger present*	90 (3%)
No trigger listed	15 (<1%)

Values are presented as a number (% of total sample) or median [IQR] or mean (SD).

*Missing information on 23 patients out of 2801 patients with blood loss concerning amount of blood loss; 71 patients concerning Hb at beginning of surgery and 1044 patients before transfusion and 791 patients at the end of surgery; 667 patients concerning INR at beginning of surgery and 2831 patients before transfusion and 2086 at the end of surgery; 927 patients concerning aPTT at beginning of surgery and 2863 patients before transfusion and 2096 patients at the end of surgery; 2215 patients concerning fibrinogen at beginning of surgery and 2929 patients before transfusion and 2406 patients at the end of surgery; 314 patients concerning platelets at the beginning of surgery, 2731 before transfusion and 1695 patients at the end of surgery; 1 patient concerning point of care device used; 4 patients concerning the information if crystalloids administered and 4 patients concerning the amount of crystalloids; 4 patients concerning the information if colloids were administered, 5 patients concerning the presence of a Hb trigger; 2 patients concerning the Hb threshold limit, 1 patient concerning the presence of a physiological trigger, 2 patients concerning the presence of a comorbidity trigger, 9 patients concerning the presence of a bleeding trigger.

Follow-up data are outlined in Table 3. 30-day follow-up data was available for 2891 patients. Patients stayed a median of 13 [7 to 26] days in hospital. Out of the 44% of patients who were admitted to the ICU, 76% had a planned ICU stay. More than half of all ICU patients were ventilated. The median ventilation time at ICU was 23 [7 to 96] hours. Out of the total sample, 70% of patients were discharged and 7% have died.

**Table 3:** Follow-up information.

	Total n = 3149	Age group 65–74 years n = 1534	Age group 75–84 years n = 1222	Age group 85–100 years n = 393
LOS until day 30; days*	13 [7 to 26]	12 [7 to 24])	13 [8 to 28]	13 [7 to 29]
Discharge until day 30*	2201 (70%)	1092 (71%)	821 (67%)	288 (73%)
Mortality until day 30*	213 (7%)	91 (6%)	92 (8%)	30 (8%)
• Units of RBCT when survived until day 30	2 [1 to 2]	2 [1 to 2]	2 [1 to 2]	2 [1 to 2]
• Units of RBCT when died until day 30	2 [1 to 4]	2 [1.5 to 4]	2 [1 to 4]	2 [1 to 3]
Admission to ICU^†^	1378 (44%)	733 (48%)	555 (45%)	90 (23%)
Admission to ICU planned	1047 (33%)	577 (38%)	406 (33%)	64 (16%)
• Units of RBCT when admission was planned	2 [1 to 3]	2 [1 to 3]	2 [1 to 2]	2 [1 to 2]
Admission to ICU unplanned	331 (11%)	156 (10%)	149 (12%)	26 (7%)
• Units of RBCT when admission was unplanned	2 [1 to 4]	2 [1 to 3]	2 [1 to 4]	2 [1 to 2.75]
Ventilation at ICU	749 (24%)	406 (26%)	297 (24%)	46 (12%)
• Hours of ventilation at ICU^‡^	23 [7 to 96]	22 [7 to 90]	24 [7 to 135]	18 [6 to 45.5]

Values are presented as a number (% of total sample or % of total age group) or median [IQR].

*Length of hospital stay, discharge and mortality until 30 days is based on available follow-up data on 2891 patients.

^†^267 patients had missing information.

^‡^1 patient had missing information.

The Cox hazard models in Table 4 are based on data of 2777 patients with complete information. They demonstrate a descriptive association between each intraoperatively administered RBCT and mortality and discharge, respectively, for the first 10 postoperative days. After adjustment for age, Hb before surgery, sex, ASA, presence of comorbidities, cancer surgery, stay in ICU, type of surgery, random intercepts for centres and additionally for blood loss, the association remained. After the first 10 postoperative days, the association was attenuated.

**Table 4:** Cox hazard models.

Association HR (95% CI) per unit of RBCT	0–10 days	>10–20 days	>20–30 days
**Units of RBCT and relative risk of discharge:**			
unadjusted	0.78 (0.74–0.83)	0.96 (0.85–1.08)	0.95 (0.84–1.08)
adjusted*	0.83 (0.78–0.88)	0.99 (0.88–1.11)	0.99 (0.88–1.11)
additionally adjusted^†^	0.82 (0.78–0.87)	0.99 (0.88–1.11)	0.98 (0.87–1.11)
**Units of RBCT and relative risk of mortality:**			
unadjusted	1.11 (1.08–1.15)	1.05 (0.94–1.17)	0.99 (0.85–1.16)
adjusted*	1.10 (1.05–1.15)	1.02 (0.90–1.15)	0.95 (0.80–1.13)
additionally adjusted^†^	1.10 (1.05–1.15)	1.02 (0.90–1.16)	0.96 (0.80–1.14)

*adjusted for age (years), Hb (g dl^-1^), sex, ASA (1 and 2; 3; 4 and 5), presence of comorbidities (yes/no), cancer surgery (yes/no), stay in ICU (no/yes-planned/yes-not planned), type pf surgery, random intercepts for centres. ^†^additionally adjusted for blood loss (<500ml, 500-1000ml, ≥1000ml).

Models are based on data of 2777 patients with complete information. Number of events (censored observations) in the three time periods concerning association between RBCT and LOS are 1028 (126), 690 (54), and 402 (477), respectively. AIC are 30740 for the unadjusted model, 30104 for the adjusted and 30108 for the additionally adjusted model. Number of events (censored observations) in the three time periods concerning the association between RBCT and mortality are 118 (1036), 49 (695), and 30 (849), respectively. AIC are 2919 for the unadjusted model, 2742 for the adjusted and 2743 for the additionally adjusted model.

## Discussion

This secondary analysis of the ETPOS trial yields the first evaluation of transfusion practices in specifically elderly surgical patients throughout Europe. We found a descriptive association between each intraoperatively administered RBCT and mortality and discharge, respectively, within the first 10 postoperative days. In our unadjusted model, the hazard ratio (HR) for mortality was 1.11 (95% CI: 1.08–1.15) and the HR for discharge was 0.78 (95% CI: 0.74–0.83). After adjustment for 10 variables, the hazard ratios were similar with a HR for mortality of 1.10 (95% CI: 1.05–1.15) and a HR for discharge of 0.82 (95% CI: 0.78–0.87).

We identified a mean Hb value of 84 (15) and a median Hb value of 83 [75 to 92] g/l prior to the first RBCT. The wide range suggests a variability in general transfusion practice, which has been demonstrated in previous studies [8, 10]. Of note, the mean Hb value at the beginning of surgery was 108 (21) g/l showing that the average patients in this study were, according to the WHO definition [24], already anaemic pre-operatively. Altogether, the patient collective in our study presented a considerably higher prevalence of anaemia than the general elderly population in the United States [2]. As our study assessed only patients who had received one or more units of RBCT, this is coherent with previous findings that pre-operative anaemia is associated with perioperative RBCT [5]. Also, anaemia in the elderly often occurs in the context of chronic diseases [26], which is reflected in the high number of patients with transfusion relevant comorbidities (62%) and classification of ≥ ASA 3 (71%) in our study. One third of the patients in this study underwent cancer-related surgery, which might be a further reason for the high prevalence of pre-operative anaemia [27]. Thus, it seems that the full potential concerning measures to optimize Hb values pre-operatively has not yet been realized. In general, guidelines recommend early diagnosis and treatment of pre-operative anaemia and useful diagnosis and treatment algorithms have been published [28–31].

Even though we analysed only patients who received at least one RBCT intraoperatively, the median blood loss of 700 [400 to 1350] ml is noteworthy, but was not further analysed in this study. Future studies should investigate which strategies to minimize intraoperative blood loss are in place and what can be further optimized. A comparison between age groups, surgical subgroups, within different countries and continents may yield valuable information.

Regarding transfusion triggers, we found that frequently more than one trigger backed the decision for RBCT with emphasis on physiological transfusion triggers. The high number of unavailable laboratory values before transfusion indicates most likely that these were not measured. Rather, anaesthesiologists substantiate the decision to administer RBCT intraoperatively on a variety of factors as shown in Table 2. Guidelines have underlined that an Hb value alone cannot replace clinical evaluation and emphasized the importance of an individual patient assessment [14, 29, 32]. Our results indicate that European intraoperative RBCT regimes for elderly patients are taking this perspective into account.

In our study, the overall 30-day mortality rate was 7%. To our knowledge, data on all cause postoperative 30-day mortality in the elderly population in Europe is scarce and subject of ongoing research (Periinterventional Outcome Study in the Elderly [POSE], NCT03152734). In the US, Hamel and colleagues found that 30-day all-cause mortality in patients ≥80 years undergoing elective as well as emergent surgery within the Veterans Affairs National Surgical Quality Improvement Project was 8%. However, the authors acknowledged that the mortality was <2% for many commonly performed procedures [33]. Another retrospective cohort study from the U.S. showed that among 239,286 American veterans, RBCT were associated with a lower 30-day mortality among elderly patients undergoing major non-cardiac surgery, if there was substantial blood loss or low preoperative haematocrit levels. However, if pre-operative haematocrit levels were elevated or blood loss was below 500 ml, mortality was increased [34]. In a retrospective analysis of more than 10,000 American surgical patients with severe anaemia, intraoperative RBCT was associated with a higher risk of mortality and morbidity, even though the reasons for these findings remained unclear [35]. The comparison of our mortality rate to previous research is hampered by the differences in surgical procedures and patient collectives as well as in follow up procedures. However, our results demonstrate that the HR for mortality after intraoperative RBCT in the investigated elderly European population is increased within the first 10 days after surgery, yet this association does not prevail after these days.

It is important to note that many other studies only evaluated 30-day mortality. Our results indicate that it may be appropriate to evaluate different time periods within the first 30 postoperative days. In general, several cohort studies reported increased mortality and LOS after RBCT [36]. Of note, in patients with cancer undergoing curative surgery, RBCT have also been associated with increased risk of death and relapse [37].

Ducrocq and colleagues found that in patients with acute myocardial infarction and anaemia a restrictive transfusion strategy was non-inferior to a liberal transfusion strategy with respect to major adverse cardiovascular events [18]. The median age of the studied population was 77 years. It was pointed out, that the confidence interval, however, included what may be a clinically important harm [18]. Mazer and colleagues found that in cardiac surgical patients aged 75 years and older, who were at moderate-to-high risk for death, a restrictive transfusion strategy was associated with a lower risk of the composite outcome than a liberal transfusion strategy [17]. Our study was not adequately designed to draw valuable conclusions for this subgroup. To date, it is subject of ongoing research why exactly RBCT have been shown to be associated with adverse outcomes. Immunomodulating effects of RBCT, lesions of red blood cells due to storage duration, and increased platelet reactivity have been discussed [36, 38].

In contrast, a recent systematic review and meta-analysis found that a restrictive transfusion regime even increased the risks of cardiovascular events irrespective of pre-existing cardiovascular disease in patients undergoing hip fracture surgery [39]. Another meta-analysis reported improved survival for perioperative adult patients receiving a liberal blood transfusion strategy [19].

Simon and colleagues evaluated nine randomized controlled trials in which a substantial proportion of patients was older than 65 years. They identified a higher 30-day mortality risk in elderly patients following a restrictive transfusion strategy than a liberal transfusion regime [11]. Concerning LOS, however, no relation to transfusion strategy was found [11]. If the older population benefits from a higher transfusion threshold is currently being investigated in a prospective multicentre trial (Liberal Transfusion Strategy in Elderly Patients [LIBERAL], NCT03369210). This study will also provide insights into how the administration of a median of 2 [1–2] units of intraoperative RBCT and the relatively high Hb value at the end of surgery in our study population should be evaluated. Given the altered physiological status in elderly patients as well as the high prevalence of comorbidities also found in our study, it is essential to conduct further research in this age group, particularly in face of the demographic changes.

Finally, it is important to keep in mind that the association between intraoperatively administered RBCT and mortality as well as discharge, found in our study, is descriptive and does not prove causality. Some scientists have pointed to confounding aspects referring to the argumentation that sicker patients receive blood transfusion more frequently, but are per se also more likely to die; and that surgical patients with relevant blood loss and hence more RBCT are more likely to have worse outcomes, accordingly [35, 40]. In order to limit for possible confounders the Cox hazards model in this study was adjusted for several variables, such as ASA and blood loss. However, there might be confounding aspects that we have not considered. E.g., it would have been desirable to address further possible confounders, such as surgical approach (open versus minimally invasive) or intraoperative complications other than massive acute bleeding, but this information was not available in the ETPOS database. Other observational studies have provided a more detailed analysis for the association between RBCT and mortality in the surgical population taking more potential confounders into consideration [34, 35]. Ultimately, randomized controlled interventional trials are needed to shed light on the causal relationship.

Even though data was originally collected prospectively, the retrospective design of this secondary analysis presents a major limitation. Our analysis relied on the data available in the closed ETPOS database, which precluded retracing of a relatively high number of patients with missing transfusion-related and follow-up information. Thus, we cannot exclude an attrition bias. Further, we were unable to verify abnormal data with former study centres.

Furthermore, information on clinically relevant outcomes such as pulmonary, cardiac, renal or cognitive status, would have been of interest, but were not investigated in the original ETPOS study. However, the presence of these postoperative complications might lead to longer LOS, which has been assessed.

A considerable disadvantage of the ETPOS study protocol was that patients were only followed up until death or discharge for a *maximum* of 30 days. Hence, information on mortality or time in hospital of patients, who died or who were readmitted after the initial discharge, was not available for this analysis.

The analysed patient cohort presents an elderly population undergoing a range of elective surgical procedures in different centres. As aging occurs at individual rates and the pre-existing conditions differ widely, older patients present a vastly inhomogeneous group [41, 42]. Thus, generally extrapolating findings to one specific patient is particularly critical in studies examining the elderly population and the importance of an individual patient evaluation cannot be emphasized enough. Noteworthy, however, the association between each intraoperative RBCT and mortality and discharge within the first 10 postoperative days persisted even after taking several individual preconditions into account.

The data for this study was collected in 2013. Within the course of the last years, PBM principles and point-of-care-testing may have been intensified and led to a decreased demand for intraoperative RBCT. Further, several randomized controlled trials, which point towards non-inferiority of a restrictive regime, have been published since then [17, 18]. Nevertheless, to our knowledge, since ETPOS, no newer study analysed intraoperative European transfusion practices. Hence, this substudy of the ETPOS dataset on specifically patients aged ≥65 years presents the only available evidence on transfusion practices and related outcomes in specifically the growing elderly surgical population in Europe. It would be of great interest for future studies to investigate the influence of PBM and point-of-care-testing on daily clinical practice and patient outcomes throughout Europe today. Interestingly, even though the data for this study was collected in 2013, important principles of today’s transfusion guidelines have already been considered. Of note, guidelines available at that time had already underlined the importance of the clinical situation of the individual patient and it seems that only a minority of anaesthesiologists in our study transfused RBCT based solely on the Hb-value whereas in most cases more than one transfusion trigger was present [43–45].

Altogether, future prospective, randomized trials are needed to further examine the effect of intraoperative RBCT on mortality and discharge from hospital in the elderly surgical population. Concerning these future studies, our results indicate that a thorough and detailed investigation of different time periods within the first 30 postoperative days is essential. Further, an assessment of differences between younger and older patients with regard to transfusions practices and outcomes is recommended. For now, transferred to daily clinical practice, our data might warrant a closer monitoring of elderly patients who received intraoperative RBCT for the first 10 postoperative days.

## Conclusion

This secondary analysis of the ETPOS trial assessed intraoperative transfusion practices and related outcomes in specifically the elderly European surgical population. We revealed a mean Hb value at the beginning of surgery of 108 (21) g/l, 84 (15) g/l before transfusion and 101 (16) g/l at the end of surgery. A median of 2 [1–2] units of RBCT were administered intraoperatively. Besides the Hb value and comorbidities, physiological transfusion triggers play a major role for intraoperative RBCT, suggesting that an individual patient evaluation was taken into account. Transferred to daily clinical practice, our results indicate that pre-operative anaemia in older surgical patients throughout Europe is undertreated. Our findings demonstrate a descriptive association between each intraoperatively administered RBCT and mortality and discharge, respectively, within the first 10 postoperative days. A closer monitoring of elderly patients receiving intraoperative RBCT for this time period might be justifiable. Further research on the causal relation between RBCT and outcomes and on optimal transfusion strategies for specifically the elderly population is warranted. A thorough time-related analysis of different time periods within the first 30 postoperative days is recommended for future studies assessing transfusion practices.

## Supporting information

S1 Checklist STROBE checklist. (DOCX)

S1 File Flow chart. (DOCX)

S1 Fig Transfusion by type of surgery. (DOCX)

## References

[1] Eurostat. Ageing Europe. Looking at the lives of older people in the EU. 2019 edition. 2019 [Cited 2020 April 27]. Available from: https://ec.europa.eu/eurostat/documents/3217494/10166544/KS-02-19%E2%80%91681-EN-N.pdf/c701972f-6b4e-b432-57d2-91898ca94893

[2] Guralnik JM, Eisenstaedt RS, Ferrucci L, Klein HG, Woodman RC. Prevalence of anemia in persons 65 years and older in the United States: evidence for a high rate of unexplained anemia. Blood. 2004;104(8):2263–8. doi: 10.1182/blood-2004-05-1812 15238427

[3] Musallam KM, Tamim HM, Richards T, Spahn DR, Rosendaal FR, Habbal A, et al. Preoperative anaemia and postoperative outcomes in non-cardiac surgery: a retrospective cohort study. Lancet. 2011;378(9800):1396–407. doi: 10.1016/S0140-6736(11)61381-0 21982521

[4] Shander A, Van Aken H, Colomina MJ, Gombotz H, Hofmann A, Krauspe R, et al. Patient blood management in Europe. Br J Anaesth. 2012;109(1):55–68. doi: 10.1093/bja/aes139 22628393 PMC3374574

[5] Khanna MP, Hebert PC, Fergusson DA. Review of the clinical practice literature on patient characteristics associated with perioperative allogeneic red blood cell transfusion. Transfus Med Rev. 2003;17(2):110–9. doi: 10.1053/tmrv.2003.50008 12733104

[6] Brown CHt, Savage WJ, Masear CG Walston JD, Tian J, Colantuoni E, et al. Odds of transfusion for older adults compared to younger adults undergoing surgery. Anesth Analg. 2014;118(6):1168–78. doi: 10.1213/ANE.0000000000000033 24413550 PMC5517014

[7] Greinacher A, Fendrich K, Brzenska R, Kiefel V, Hoffmann W. Implications of demographics on future blood supply: a population-based cross-sectional study. Transfusion. 2011;51(4):702–9. doi: 10.1111/j.1537-2995.2010.02882.x 20849411

[8] Qian F, Osler TM, Eaton MP, Dick AW, Hohmann SF, Lustik SJ, et al. Variation of blood transfusion in patients undergoing major noncardiac surgery. Ann Surg. 2013;257(2):266–78. doi: 10.1097/SLA.0b013e31825ffc37 22801086

[9] Griffiths R, Beech F, Brown A, Dhesi J, Foo I, Goodall J, et al. Peri-operative care of the elderly 2014: Association of Anaesthetists of Great Britain and Ireland. Anaesthesia. 2014;69 Suppl 1:81–98. doi: 10.1111/anae.12524 24303864

[10] Frank SM, Savage WJ, Rothschild JA, Rivers RJ, Ness PM, Paul SL, et al. Variability in blood and blood component utilization as assessed by an anesthesia information management system. Anesthesiology. 2012;117(1):99–106. doi: 10.1097/ALN.0b013e318255e550 22531332

[11] Simon GI, Craswell A, Thom O, Fung YL. Outcomes of restrictive versus liberal transfusion strategies in older adults from nine randomised controlled trials: a systematic review and meta-analysis. Lancet Haematol. 2017;4(10):e465–e74. doi: 10.1016/S2352-3026(17)30141-2 28919087

[12] Goodnough LT, Shander A. Patient blood management. Anesthesiology. 2012;116(6):1367–76. doi: 10.1097/ALN.0b013e318254d1a3 22487863

[13] Goodnough LT, Levy JH, Murphy MF. Concepts of blood transfusion in adults. Lancet. 2013;381(9880):1845–54. doi: 10.1016/S0140-6736(13)60650-9 23706801

[14] Carson JL, Guyatt G, Heddle NM, Grossman BJ, Cohn CS, Fung MK, et al. Clinical Practice Guidelines From the AABB: Red Blood Cell Transfusion Thresholds and Storage. JAMA. 2016;316(19):2025–35. doi: 10.1001/jama.2016.9185 27732721

[15] NICE. NICE guideline [NG24] Blood transfusion. 2015 [Cited 2020 May 5]. Available from: https://www.nice.org.uk/guidance/ng24/chapter/Recommendations#red-blood-cells-2 32813480

[16] Practice guidelines for perioperative blood management: an updated report by the American Society of Anesthesiologists Task Force on Perioperative Blood Management. Anesthesiology. 2015;122(2):241–75. doi: 10.1097/ALN.0000000000000463 25545654

[17] Mazer CD, Whitlock RP, Fergusson DA, Hall J, Belley-Cote E, Connolly K, et al. Restrictive or Liberal Red-Cell Transfusion for Cardiac Surgery. N Engl J Med. 2017;377(22):2133–44. doi: 10.1056/NEJMoa1711818 29130845

[18] Ducrocq G, Gonzalez-Juanatey JR, Puymirat E, Lemesle G, Cachanado M, Durand-Zaleski I, et al. Effect of a Restrictive vs Liberal Blood Transfusion Strategy on Major Cardiovascular Events Among Patients With Acute Myocardial Infarction and Anemia: The REALITY Randomized Clinical Trial. JAMA. 2021;325(6):552–60. doi: 10.1001/jama.2021.0135 33560322 PMC7873781

[19] Fominskiy E, Putzu A, Monaco F, Scandroglio AM, Karaskov A, Galas FR, et al. Liberal transfusion strategy improves survival in perioperative but not in critically ill patients. A meta-analysis of randomised trials. Br J Anaesth. 2015;115(4):511–9. doi: 10.1093/bja/aev317 26385661

[20] Docherty AB, O’Donnell R, Brunskill S, Trivella M, Doree C, Holst L, et al. Effect of restrictive versus liberal transfusion strategies on outcomes in patients with cardiovascular disease in a non-cardiac surgery setting: systematic review and meta-analysis. BMJ. 2016;352:i1351. doi: 10.1136/bmj.i1351 27026510 PMC4817242

[21] Murphy GJ, Pike K, Rogers CA, Wordsworth S, Stokes EA, Angelini GD, et al. Liberal or restrictive transfusion after cardiac surgery. N Engl J Med. 2015;372(11):997–1008. doi: 10.1056/NEJMoa1403612 25760354

[22] Meier J, Filipescu D, Kozek-Langenecker S, Llau Pitarch J, Mallett S, Martus P, et al. Intraoperative transfusion practices in Europe. Br J Anaesth. 2016;116(2):255–61. doi: 10.1093/bja/aev456 26787795 PMC4718146

[23] Meier J, Filipescu D, Kozek-Langenecker S, Llau Pitarch J, Mallett S, Martus P, et al. ETPOS Protocol. 2013 [Cited 2020 April 27]. Available from: https://www.esahq.org/research/clinical-trial-network/published-trials/etpos/protocol/10.1097/EJA.0b013e32835f0052 23571430

[24] World Health Organization. Haemoglobin concentrations for the diagnosis of anaemia and assessment of severity. 2011 [Cited 2020 September 4). Available from: https://www.who.int/vmnis/indicators/haemoglobin.pdf.

[25] Ripatti S, Palmgren J. Estimation of multivariate frailty models using penalized partial likelihood. Biometrics. 2000;56(4):1016–22. doi: 10.1111/j.0006-341x.2000.01016.x 11129456

[26] Balducci L. Epidemiology of anemia in the elderly: information on diagnostic evaluation. J Am Geriatr Soc. 2003;51(3 Suppl):S2–9. doi: 10.1046/j.1532-5415.51.3s.4.x 12588565

[27] Khan FA, Shukla AN, Joshi SC. Anaemia and cancer treatment: a conceptual change. Singapore Med J. 2008;49(10):759–64. 18946607

[28] Goodnough LT, Maniatis A, Earnshaw P, Benoni G, Beris P, Bisbe E, et al. Detection, evaluation, and management of preoperative anaemia in the elective orthopaedic surgical patient: NATA guidelines. Br J Anaesth. 2011;106(1):13–22. doi: 10.1093/bja/aeq361 21148637 PMC3000629

[29] Mueller MM, Van Remoortel H, Meybohm P, Aranko K, Aubron C, Burger R, et al. Patient Blood Management: Recommendations From the 2018 Frankfurt Consensus Conference. JAMA. 2019;321(10):983–97. doi: 10.1001/jama.2019.0554 30860564

[30] Muñoz M, Acheson AG, Auerbach M, Besser M, Habler O, Kehlet H, et al. International consensus statement on the peri-operative management of anaemia and iron deficiency. Anaesthesia. 2017;72(2):233–47. doi: 10.1111/anae.13773 27996086

[31] Kozek-Langenecker SA, Ahmed AB, Afshari A, Albaladejo P, Aldecoa C, Barauskas G, et al. Management of severe perioperative bleeding: guidelines from the European Society of Anaesthesiology: First update 2016. Eur J Anaesthesiol. 2017;34(6):332–95. doi: 10.1097/EJA.0000000000000630 28459785

[32] National Blood Authority. Patient blood management guidleines: module 2; perioperative. 2012 [Cited 2020 May 12]. Available from: https://www.blood.gov.au/system/files/documents/pbm-module-2.pdf.

[33] Hamel MB, Henderson WG, Khuri SF, Daley J. Surgical outcomes for patients aged 80 and older: morbidity and mortality from major noncardiac surgery. J Am Geriatr Soc. 2005;53(3):424–9. doi: 10.1111/j.1532-5415.2005.53159.x 15743284

[34] Wu WC, Smith TS, Henderson WG, Eaton CB, Poses RM, Uttley G, et al. Operative blood loss, blood transfusion, and 30-day mortality in older patients after major noncardiac surgery. Ann Surg. 2010;252(1):11–7. doi: 10.1097/SLA.0b013e3181e3e43f 20505504

[35] Glance LG, Dick AW, Mukamel DB, Fleming FJ, Zollo RA, Wissler R, et al. Association between intraoperative blood transfusion and mortality and morbidity in patients undergoing noncardiac surgery. Anesthesiology. 2011;114(2):283–92. doi: 10.1097/ALN.0b013e3182054d06 21239971

[36] Marik PE, Corwin HL. Efficacy of red blood cell transfusion in the critically ill: a systematic review of the literature. Crit Care Med. 2008;36(9):2667–74. doi: 10.1097/CCM.0b013e3181844677 18679112

[37] Petrelli F, Ghidini M, Ghidini A, Sgroi G, Vavassori I, Petrò D, et al. Red blood cell transfusions and the survival in patients with cancer undergoing curative surgery: a systematic review and meta-analysis. Surg Today. 2021. doi: 10.1007/s00595-020-02192-3 33389174

[38] Silvain J, Abtan J, Kerneis M, Martin R, Finzi J, Vignalou JB, et al. Impact of red blood cell transfusion on platelet aggregation and inflammatory response in anemic coronary and noncoronary patients: the TRANSFUSION-2 study (impact of transfusion of red blood cell on platelet activation and aggregation studied with flow cytometry use and light transmission aggregometry). J Am Coll Cardiol. 2014;63(13):1289–96. doi: 10.1016/j.jacc.2013.11.029 24361322

[39] Gu WJ, Gu XP, Wu XD, Chen H, Kwong JSW, Zhou LY, et al. Restrictive Versus Liberal Strategy for Red Blood-Cell Transfusion: A Systematic Review and Meta-Analysis in Orthopaedic Patients. J Bone Joint Surg Am. 2018;100(8):686–95. doi: 10.2106/JBJS.17.00375 29664857

[40] Carson JL, Reynolds RC, Klein HG. Bad bad blood? Crit Care Med. 2008;36(9):2707–8. doi: 10.1097/CCM.0b013e3181843ee7 18728495

[41] Bailes BK. Perioperative care of the elderly surgical patient. AORN J. 2000;72(2):186–207; quiz 18–21, 23, 25–6. doi: 10.1016/s0001-2092(06)61931-5 10957942

[42] Brummel-Smith K, Gunderson A. Caring for older patients and an aging population. In: Ham R, Sloane P, Bernard M, editors. Primary care geriatrics: a case-based approach. Philadephia: MOSBY Elsevier; 2007. p. 3.

[43] Practice Guidelines for blood component therapy: A report by the American Society of Anesthesiologists Task Force on Blood Component Therapy. Anesthesiology. 1996;84(3):732–47. 8659805

[44] Carson JL, Grossman BJ, Kleinman S, Tinmouth AT, Marques MB, Fung MK, et al. Red blood cell transfusion: a clinical practice guideline from the AABB*. Ann Intern Med. 2012;157(1):49–58. doi: 10.7326/0003-4819-157-1-201206190-00429 22751760

[45] Murphy MF, Wallington TB, Kelsey P, Boulton F, Bruce M, Cohen H, et al. Guidelines for the clinical use of red cell transfusions. Br J Haematol. 2001;113(1):24–31. doi: 10.1046/j.1365-2141.2001.02701.x 11328275

