# Supplementary material for: Intraoperative transfusion practices and perioperative outcome in the European elderly: A secondary analysis of the observational ETPOS study
Source: PLoS One. 2022 Jan 4;17(1):e0262110. doi: 10.1371/journal.pone.0262110 (PMC8726458; doi:10.1371/journal.pone.0262110)
Supplement: S1 File — (DOCX) [file pone.0262110.s002.docx]

**Supplementary S 2**

**Flow chart**


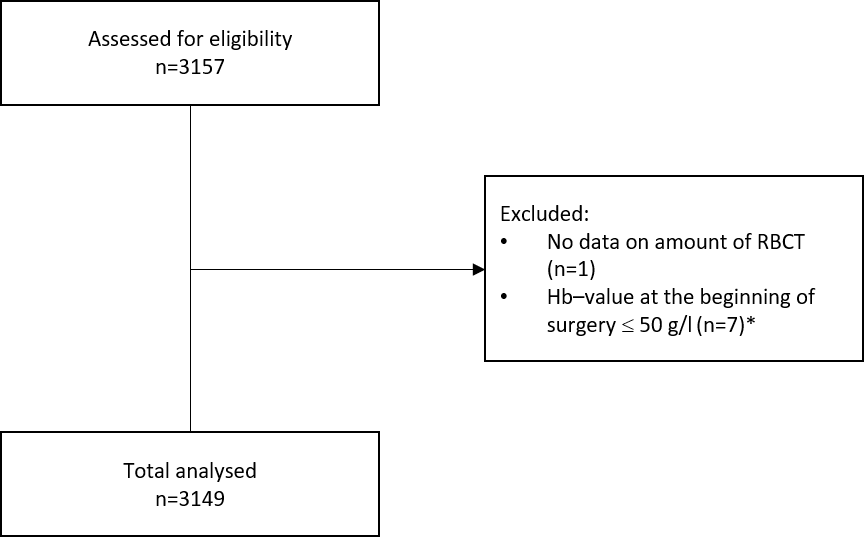


* Patients with extremely low Hb value <50 g/l prior to surgery were excluded from analysis as this contradicts the principle of an elective surgery
