# Supplementary figures and images for: Intraoperative transfusion practices and perioperative outcome in the European elderly: A secondary analysis of the observational ETPOS study
Source: PLoS One. 2022 Jan 4;17(1):e0262110. doi: 10.1371/journal.pone.0262110 (PMC8726458; doi:10.1371/journal.pone.0262110)

**Supplementary S 3**

**Transfusion by type of surgery**


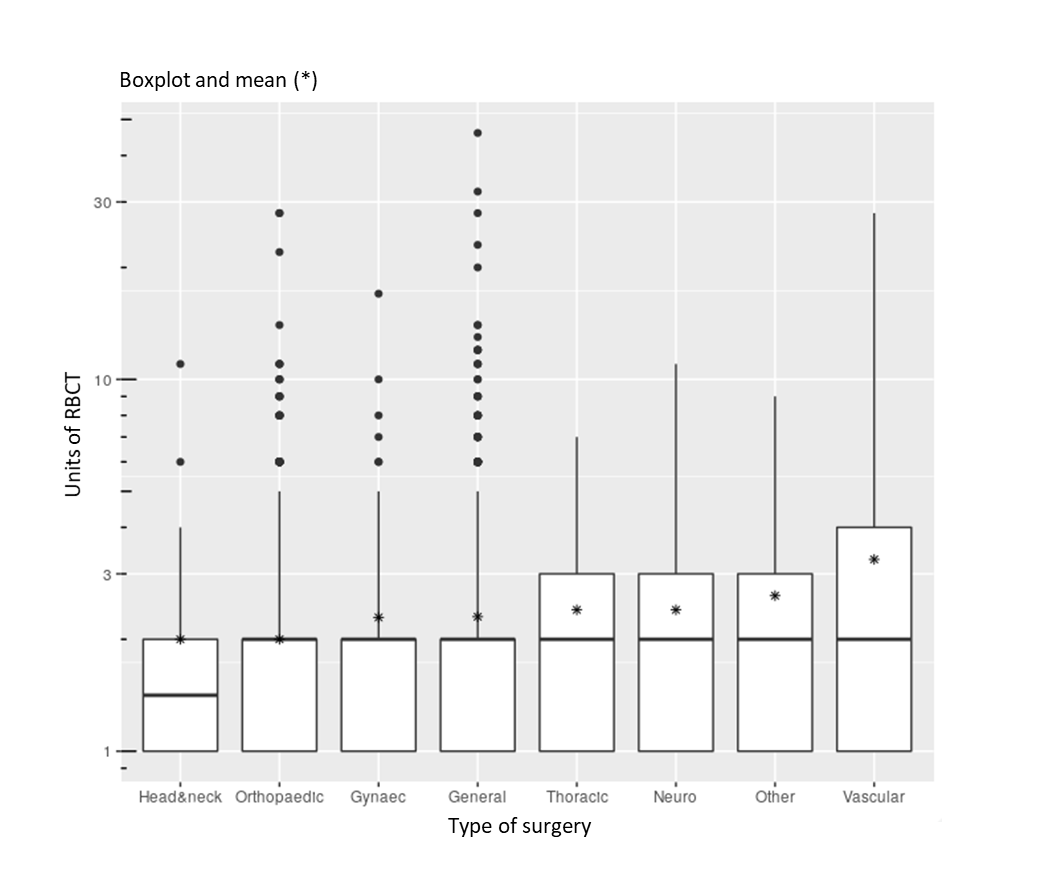

Supplement: S1 Fig — (DOCX) [file pone.0262110.s003.docx]
